# Suppression of the inflammatory response in experimental arthritis is mediated via estrogen receptor α but not estrogen receptor β

**DOI:** 10.1186/ar3032

**Published:** 2010-05-24

**Authors:** John Dulos, Peter Vijn, Cindy van Doorn, Claudia L Hofstra, Desiree Veening-Griffioen, Jan de Graaf, Fred A Dijcks, Annemieke MH Boots

**Affiliations:** 1 Schering-Plough Research Institute, PO box 20, 5340 BH Oss, The Netherlands

## Abstract

**Introduction:**

The immune modulatory role of estrogens in inflammation is complex. Both pro- and anti-inflammatory effects of estrogens have been described. Estrogens bind both estrogen receptor (ER)α and β. The contribution of ERα and ERβ to ER-mediated immune modulation was studied in delayed type hypersensitivity (DTH) and in experimental arthritis

**Methods:**

ER-mediated suppression of rat adjuvant arthritis (AA) was studied using ethinyl-estradiol (EE) and a selective ERβ agonist (ERB-79). Arthritis was followed for 2 weeks. Next, effects of ER agonists (ethinyl-estradiol, an ERα selective agonist (ERA-63) and a selective ERβ agonist (ERB-79) on the development of a tetanus toxoid (TT)-specific delayed type hypersensitivity response in wild type (WT) and in ERα - or ERβ-deficient mice were investigated. Finally, EE and ERA-63 were tested for their immune modulating potential in established collagen induced arthritis in DBA/1J mice. Arthritis was followed for three weeks. Joint pathology was examined by histology and radiology. Local synovial cytokine production was analyzed using Luminex technology. Sera were assessed for COMP as a biomarker of cartilage destruction.

**Results:**

EE was found to suppress clinical signs and symptoms in rat AA. The selective ERβ agonist ERB-79 had no effect on arthritis symptoms in this model. In the TT-specific DTH model, EE and the selective ERα agonist ERA-63 suppressed the TT-specific swelling response in WT and ERβKO mice but not in ERαKO mice. As seen in the AA model, the selective ERβ agonist ERB-79 did not suppress inflammation. Treatment with EE or ERA-63 suppressed clinical signs in collagen induced arthritis (CIA) in WT mice. This was associated with reduced inflammatory infiltrates and decreased levels of proinflammatory cytokines in CIA joints.

**Conclusions:**

ERα, but not ERβ, is key in ER-mediated suppression of experimental arthritis. It remains to be investigated how these findings translate to human autoimmune disease.

## Introduction

It is well known that many autoimmune diseases are more prevalent in women than in men [1]. More specifically, rheumatoid arthritis (RA) is often diagnosed in the childbearing years when both onset and exacerbations are associated with the post-partum period, and pregnancy is associated with milder disease symptoms [2,3]. In addition, RA incidence peaks in the postmenopausal state associated with a drop in endogenous estrogen levels [4,5]. These early findings suggested an important role for female sex hormones in chronic inflammatory disease.

Animal models have been widely used to study the role of female sex hormones in inflammation. Ovariectomy-induced loss of endogenous estrogen production in female DBA/1 mice increased arthritic signs in collagen-induced arthritis (CIA) [6]. Female mice, similar to the human situation, show pregnancy-associated protection of joint disease with post-partum flares of arthritis [7]. The post-partum flare seen in CIA was suppressed by exogenous administration of ethinyl-estradiol (EE) but not with progesterone and hydrocortisone [7]. Levels of EE that were suppressive were comparable with estrogen levels seen at pregnancy. Similar results have now been reported for estrogens in experimental autoimmune models such as experimental autoimmune encephalomyelitis (EAE) and experimental AA [8-10]. In contrast, estrogen was found to accelerate autoimmune phenomena in experimental systemic lupus erythromatosus (SLE) [11].

Estrogens mediate their immune modulatory effects via classical estrogen receptors (ERs) [12]. Cloning of ERα was first reported in 1986 [13]. Ten years later a second receptor was identified in mice, rats and humans, and was named ERβ [14-16]. ER expression has been described in various cell types involved in inflammatory processes including T cells, B cells, dendritic cells, monocytes and macrophages [17-19]. Differential expression of the ER subtypes in different cell types and in different microenvironments may thus impact estrogen-mediated effects [20]. Interestingly, relatively high ERβ expression levels were observed in synovial tissue of RA patients; ERβ dominant expression was reported in synovial fibroblasts, inflammatory cells and in the synovial lining layer [21,22]. The data suggest an inflammation-dependent upregulation of ERβ relative to ERα in RA.

In experimental arthritis, most studies report an immune suppressive effect of E2 or EE, which bind both ERs in an agonistic mode [23,24]. This finding is in line with an earlier onset of arthritis in mice when treated with the ERα/β antagonist ICI 182780 [10]. Harris and colleagues have reported ERβ-mediated suppression of inflammation in rat adjuvant arthritis and in the HLA-B27 transgenic rat model of inflammatory bowel disease using the ERβ selective agonist ERB-041 [25].

Here, we chose to investigate the relative contribution of ERα or ERβ to ER-mediated immune-suppression *in vivo*. To this end, both selective ERα and ERβ agonistic compounds and ERα-and ERβ-deficient mice were used. First, ER-mediated immune modulation was evaluated in lewis rat adjuvant arthritis. Second, we investigated ER-mediated suppression of the tetanus-toxoid (TT)-specific delayed type hypersensitivity (DTH) in wild type, ERα-deficient and ERβ-deficient mice. Finally, EE and a selective ERα agonist compound were evaluated in an established CIA. Our data show an important role for ERα but not ERβ in suppression of inflammatory processes in experimental arthritis.

## Materials and methods

### Mice and rats

All the experiments were approved by the Animal Welfare Committee of Schering-Plough, Oss, The Netherlands.

### Pharmacokinetics of EE, ERA-63 and ERB-79

Due to the poor oral bioavailability of estrogens, treatment in most of the animal models described so far involved the use of estrogen injections or implantation of estrogens such as E2. We used the synthetic estrogen EE, synthesized in house, at dosages that have been described to be orally effective in the treatment of EAE and CIA [23,26] The pharmacologic properties for the ERα agonist ERA-63 (Org 37663) have been described previously showing efficacy in inflammatory models at 1.5 mg/kg [27]. For both EE and ERA-63, the increase in uterus weight can be considered a pharmacodynamic marker of estrogenic activity *in vivo *[28]. Pharmacologic characterization of the selective ERβ agonist ERB-79 in rats has recently been reported [29]. ERB-79 is an ERβ agonist displaying a more than 484-fold selectivity over ERα based on *in vitro *ERα transactivation and ERβ transactivation assays with EC50 values of 7.9 × 10^-8 ^M (potency of 0.03% relative to E2) versus 4.48 × 10^-10 ^M (potency of 14.52% relative to E2), respectively. The compound has no ERα or ERβ antagonistic properties.

In order to arrive at a dose of ERB-79 engaging ERβ but not ERα in mice, an *in vivo *titration for ERα activity was performed. To that end, female DBA/1J mice were ovariectomized and treated, daily, by subcutaneous injection, for 21 days with EE (0.025 mg/kg) or ERB-79 at a dose of 1 mg/kg, 3 mg/kg or 10 mg/kg. Next, uteri were dissected free, weighted and thereafter processed for histological examination. ERB-79 increased the more ERα sensitive marker of epithelial cell height at 3 mg/kg or more ERB-79 (Table 1a). However, at that dose no ERα- mediated effect was seen on uterus weight. In the present study we thus chose a dose of 3 mg/kg subcutaneous yielding plasma level concentrations (around 1 × 10^-8 ^M) adequate for engaging ERβ but unlikely to engage ERα.

**Table 1:** Effect of EE and ERB-79 on uterus weight and luminal epithelial height

	Dose	Uterus weight (mg: mean ± SD)	Uterus Luminal epithelial height (μM: mean ± SD)
Vehicle sc	-	14.6 ± 9.5	11.7 ± 1.1
EE sc	0.025	107.6 ± 20.2*	28.5 ± 1.8*
ERB-79 sc	1	9.7 ± 2.5	11.8 ± 1.1
ERB-79 sc	3	14.2 ± 2.7	13.7 ± 1.5*
ERB-79 sc	10	18.2 ± 4.2	14.1 ± 0.8*

After ovariectomy of female DBA/1J mice, mice were treated daily subcutaneaously, for 21 days with vehicle (0.5% gelatin and 5% mannitol in water), the estrogen receptor (ER)α/β agonist ethinyl estradiol (EE; 0.025 mg/kg) or the ERβ agonist ERB-79 at 1 mg/kg, 3 mg/kg and 10 mg/kg (n = 8 mice per treatment group). Uterus was dissected free, weighted and thereafter fixed in 4% formaldehyde. Uterus was embedded in paraffin and sections were stained with hematoxylin and eosin. Microscopical images are captured and measured at 125-fold magnification using an automated image analysis system (UltraSpark, Iduna Elektronics BV, Veghel, The Netherlands). Of each mouse at least two cross sections and two fields per section are measured. The mean epithelial height is measured over a stretch of luminal epithelium that is clearly transversely cut. All measurements per mouse are used to calculate one mean Luminal Epithelial Height (LEH) value for that animal. (* *P *≤ 0.05: Statistical analysis was performed using one-way analysis of variance). sc, subcutaneous; SD, standard deviation.

### Lewis rat adjuvant-induced arthritis

The study investigating effects of EE and ERB-79 on modulation of rat AA were carried out according to a standardized protocol. In brief, male Lewis rats were immunized by subcutaneous injection in the tail base with 0.1 ml (1 mg) *Mycobacterium tuberculosis *in complete Freund's Adjuvant (Difco Lab. Detroit, IL, USA). Rats (n = 8 per treatment group) were left untreated or were treated subcutaneously once daily with vehicle (gelatin 0.5%-mannitol 5% in water), dexamethasone (1 mg/kg), EE (2.5 mg/kg) or the selective ERβ agonist ERB-79 (3 mg/kg). Treatment started on day 10 when the first signs of disease activity were observed. Rats were evaluated daily (once during weekends) for arthritis severity using a macroscopic scoring system of 0 to 4 (0 = no signs of arthritis, 0.5 = partial limping/unloading of paw, 1 = redness of the paw and inability to fully stretch ankle joint, 2 = moderate swelling and redness of paw, 3 = severe redness and swelling of entire paw including digits, 4 = maximally inflamed paw, multiple joints involved). For each rat, the cumulative score was calculated by adding the scores obtained from day 0 to day 24 and presented as the mean ± standard error of the mean (n = 8 rats per group). Statistical analysis was performed using analysis of variance (ANOVA) followed by *post hoc *Least Significant Difference (LSD) test (*** *P *≤ 0.001).

### Tetanus-toxoid-induced footpad swelling

In order to exclude a major source of endogenous estrogen production, female C57bl/6 mice of 8 to 10 weeks of age were bilaterally ovariectomized under anesthesia. During a recovery period of about one week vaginal smears were taken daily to record the phase of the estrous cycle and only animals devoid of cyclic activity were included in the experiment. In all DTH experiments, animals were immunized at day 0 with 50 μl TT mixed in dimethyl dioctadecyl ammonium bromide (37.5 Lf TT/ml of dimethyl dioctadecyl ammonium bromide) intradermally in the back, just below the neck, at two different sites (2 × 50 ul). At day 7, animals were challenged intradermally with 50 μl TT mixed in Al(OH)_3 _(50 Lf TT in 1 mg/ml Al(OH)_3_) in the left footpad (ventral side). The right control footpad received vehicle only. Twenty-four hours later the left and right hind footpad thickness was measured with a micrometer designed in-house and the Δmm of antigen-specific footpad swelling was calculated according to the following formula: ((swelling left (mm) minus swelling right (mm)). At autopsy (48 hours later) the uterus was removed and weighted.

WT C57bl/6 (n = 8 per group) mice were ovariectomized and treated once daily orally with the selective ERα agonist ERA-63 (6 mg/kg), EE (0.025, 0.25 and 2.5 mg/kg) or vehicle (0.5% gelatin- 5% mannitol in water) only from day -1 to day 9. The selective ERβ agonist ERB-79 (3 mg/kg) was administered subcutaneously and compared with vehicle (subcutaneous).

Further DTH validation experiments were performed with ERα - and ERβ-deficient mice. ERαKO mice were obtained from Iafrati [30]. The ERβKO mice were generated in-house and fully characterized [31]. ERαKO, ERβKO and WT were ovariectomized and treated once daily orally with the selective ERα agonist ERA-63 (6 mg/kg) or vehicle (0.5% gelatin and 5% mannitol in water) from day -1 to day 9. The DTH response was assessed as before.

### Therapeutic murine collagen-induced arthritis

The murine CIA model was performed as described [32]. In brief, male DBA1/J mice were obtained from Bomholtgard (Ry, Denmark). Animals were housed and maintained at 23°C with water and food *ad libitum*. Mice were immunized at the base of the tail at day 0 (at the age of eight weeks) with 100 μg bovine type II collagen in complete Freund's adjuvant enriched with 2 mg/ml *M. tuberculosis *(H37Ra). Three weeks after immunization (at day 21) the animals were boosted with an intra-peritoneal injection of 100 μg collagen type II, dissolved in saline. After disease onset, animals with an arthritis score ranging from 0.25 and 1.25 were divided into separate groups of 12 mice so that the mean arthritis score of all groups was comparable at the start of treatment (day 0). Mice were considered to have arthritis when significant changes in redness and/or swelling were noted in the digits or in other parts of the paws. Arthritic animals were treated orally once daily for a period of 21 days with 0.025, 0.25 or 2.5 mg/kg EE in vehicle (0.5% gelatin and 5% mannitol in water), 0.75, 1.5 or 3 mg/kg Erα-agonist ERA-63 in vehicle, or vehicle alone. As a positive control for suppression of arthritis, animals were treated orally with 1.5 or 3 mg/kg prednisolone in vehicle. All experimental treatments were conducted in a blinded fashion. The clinical severity of arthritis (arthritis score) was graded (a scale of 0 to 2 for each paw). Mice were scored on alternative days, resulting in mean scores with a maximum of 2 for each paw, and an overall maximum of 8 per animal. To assess the effects of treatment, the area under the curve (AUC) of mean arthritis score of each animal with baseline correction (subtracting baseline AUC of arthritis score on day 0) was used. At the end of the experiment (21 days of treatment) knee synovial biopsies, hind paws and serum samples were obtained. Hind paws were evaluated using X-ray analysis [33] to assess bone destruction. X-ray photographs were examined with a Faxitron X-ray MX-20 (0.02 mm resolution) and bone destruction was scored on a scale from 0 to 5 ranging from no damage to complete destruction [34]. For histopathological analysis (infiltration and cartilage destruction) knee joints were fixed in 4% formaldehyde, decalcified in 5% formic acid and processed and evaluated as described [33]. Hematoxylin and eosin-stained sections (7 μM) were used to study joint inflammation. The severity of inflammation in joints was scored on a scale of 0 to 3 (0 = no cells, 1 = mild cellularity, 2 = moderate cellularity and 3 = maximal cellularity). To study proteoglycan depletion from the cartilage matrix, sections were stained with safranin O. Depletion of proteoglycan was scored on an arbitrary scale of 0 to 3 ranging from normal fully stained cartilage to destained cartilage.

To analyze cytokine levels with Luminex (Bio-rad, Hercules, CA, USA) technology, knee synovial biopsies were isolated as described [33], frozen in liquid nitrogen and stored at -70°C until use (see section cytokine and chemokine protein levels by Luminex).

### Cytokine and chemokine protein detection in CIA synovial tissue

To investigate the presence of cytokines/chemokines produced locally, knee synovial biopsy samples were isolated from vehicle and estrogen-treated mice. Knee synovial biopsy samples were pooled (n = 6 per treatment group), weighed and cut into small pieces with a scissor. Lysis solution was added containing 100 mmol/L potassium phosphate (PH 7.8), 0.2% Triton X-100, 1 mmol/L dithiothreitol and 1 mM protease inhibitor (prefabloc from Boehringer, Mannheim, Germany). The volume of lysis buffer was adjusted to 250 mg of tissue per ml. After the lysis buffer was added, the samples were placed on ice for 15 minutes and thereafter centrifuged for 30 minutes at 500 g. The amount of protein in the supernatant was determined using the BCA assay and the samples were aliquoted and stored at -70°C until use.

For simultaneous detection of 18 cytokines in one sample we used the Bio-Plex/Luminex mouse cytokine 18-plex panel kit, which includes antibody-conjugated beads, detection antibody and standards for detection of IL-1α, IL-1β, IL-2, IL-3, IL-4, IL-5, IL-6, IL-10, IL-12p40, IL-12p70, IL-17, Granulocyte-Colony Stimulating Factor (G-CSF), Granylocyte Macrophage Colony stimulating factor (GM-CSF), interferon (IFN)γ, the murine IL-8 homoloque KC, Macrophage Inhibitory Protein-1 (MIP-1α), Chemokine (C-C motif) ligand 5 also known as CCL5 or RANTES and TNFα using a 96-well round-bottomed micro titer plate as described by the manufacturer (Biorad, Hercules, CA, USA). Pooled supernatants from knee biopsy samples were diluted once in assay dilutent. Samples were incubated for 30 minutes on ice with antibody-conjugated beads, washed and thereafter incubated for 30 minutes with biotinylated antibody. After washing, streptavidin-PE was added and incubated for 10 minutes. The Bioplex-protein assay reader from Luminex was used. The amount (pg) of cytokine/chemokine per mg protein (pg/mg) was calculated.

### Statistical analysis

All statistics were performed using SAS. TT-DTH data were analyzed with ANOVA on factors treatment and strain (wild type and knockout) and interaction between treatment and strain. Comparisons with vehicle treatment was performed. Cartilage Oligomeric Matrix Protein (COMP) and cytokine/chemokine levels were analyzed with the Mann-Whitney U test (two-tailed) whereby treatment is compared with vehicle. Arthritis scores were analyzed with ANOVA. Estrogen- or prednisolone-treated groups were compared with vehicle (* *P *< 0.05, ** *P *< 0.01, *** *P *< 0.001) using the LSD *post hoc *comparison test.

## Results

### EE but not ERB-79 suppresses lewis rat AA

**Figure 1:**
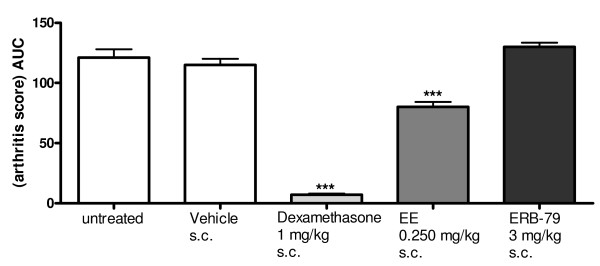
**No role for ERβ in modulation of rat AA**. Male lewis rats were injected with complete freund's adjuvant containing mycobacterium tuberculosis. Daily treatment (subcutaneous) with dexamethasone (1 mg/kg), ethinyl estradiol (EE at 2.5 mg/kg), ERB-79 (3 mg/kg) or vehicle (0.5% gelatin- 5% mannitol) started when the first signs of arthritis were observed (day 10). Animals (n = 8 per group) were scored daily for arthritis. Statistical analysis was performed using analysis of variance followed by *post hoc *Least Significant Difference (LSD)test (*** *P *≤ 0.001). AUC, area under the curve. ER, estrogen receptor.

Previously, ERβ-mediated suppression of inflammation in Lewis rat AA was reported [25]. This prompted us to study the effects of EE and our selective ERB-79 in rat AA. The ERB-79 dose of 3 mg/kg was chosen on the basis of prior studies showing ERβ but not ERα engagement [29]. EE at a dose of 0.25 mg/kg when administered subcutaneously significantly suppressed the arthritis score in this model as assessed by the AUC (Figure 1). The inhibition of inflammation by EE was partial. Dexamethasone, the positive treatment control, was able to suppress inflammation completely. Interestingly, the selective ERB-79 when dosed at 3 mg/kg subcutaneously did not suppress clinical signs of arthritis in this model. In addition, arthritis incidence and onset in ERB-79-treated animals was not affected (data not shown). The data imply that estrogen-mediated suppression in rat AA is ERα mediated.

### The estrogen-mediated suppression of the TT-DTH in wild type mice is dependent on ERα

Next, we assessed the relative contribution of ERα and ERβ to ER-mediated immune modulation in the mouse. To this end, the TT-DTH model was used in ovariectomized mice. Mice were treated with the ERα-agonist ERA-63, the ERβ-agonist ERB-79 or the non-selective estrogen EE whereby the ERα-sensitive uterus weight was used as a pharmacodynamic readout control. For ERB-79, the more sensitive marker of epithelial cell height was used to establish a dose range securing ERβ but not ERα activity (3 mg/kg; Table 1).

As expected, the glucocorticoid dexamethasone and ERB-79, at a pharmacologic defined concentration, engaged ERβ but not ERα, showed no effect on uterus weights (Table 2). The glucocorticoid dexamethasone, which is used as a positive anti-inflammatory control, strongly inhibited the TT-specific footpad swelling (Table 2). Also, a significant suppressive effect of oral treatment with EE (at both 0.25 mg/kg to 2.5 mg/kg) on TT-specific footpad swelling was observed (Table 2). Interestingly, ERA-63 dose-dependently decreased the TT-specific response whereas treatment with the ERβ-agonist ERB-79 had no effect on TT-specific swelling.

**Table 2:** Suppression of the tetanus-specific DTH response is ERα-mediated

	Δmm swelling	Uterus weight (mg)
Vehicle po	1.2 ± 0.4	19.9 ± 7.0
Dexamethasone		
3 mg/kg po	0.0 ± 0.0*	29.7 ± 17.4
EE 0.025 mg/kg po	0.7 ± 0.4	109.5 ± 23.8*
EE 0.25 mg/kg po	0.4 ± 0.2*	91.1 ± 14.4*
EE 2.5 mg/kg po	0.6 ± 0.3*	84.8 ± 17.1*
Vehicle po	1.1 ± 0.4	11.1 ± 1.6
Dexamethasone		
3 mg/kg po	0.0 ± 0.0*	14.7 ± 3.5
ERA-63 1.5 mg/kg po	0.7 ± 0.3*	136.6 ± 19.9*
ERA-63 3 mg/kg po	0.5 ± 0.2*	113.6 ± 15.4*
ERA-63 6 mg/kg po	0.4 ± 0.2*	92.1 ± 14.5*
Vehicle sc	1.3 ± 0.1	13.4 ± 0.8
EE 0.25 mg/kg sc	0.5 ± 0.1*	148.8 ± 6.4*
ERB-79 3 mg/kg sc	1.1 ± 0.2	14.8 ± 1.8

Mice (n = 8 per group) were ovariectomized at day -14. Animals were treated daily for 10 days (day -1 to day 9) with ethinyl estradiol (EE) (0.025, 0.25 and 2.5 mg/kg), ERA-63 (1.5, 3 and 6 mg/kg), the positive control dexamethasone (3 mg/kg) or vehicle (0.5% gelatin and 5% mannitol in water). ERB-79 was dosed subcutaneously at 3 mg/kg per day (2 × 1.5 mg/kg). Animals were immunized at day 0 and boosted at day 7. Twenty four hours after challenge the footpad swelling was measured. Forty eight hours after challenge mice were euthanized and the uterus was dissected and weighed. Statistical analysis was performed with analysis of variance. * *P *< 0.05. DTH, delayed type hypersensitivity; ER, estrogen receptor; po, orally; sc, subcutaneous.

To study whether estrogens modulate the antigen-specific humoral immune response, sera from estrogen-treated mice were assayed for TT-specific antibodies using an ELISA. TT-specific IgG1 titers were clearly suppressed following treatment with dexamethasone and were minimally affected following treatment with either EE or ERα-agonist ERA-63 (data not shown). Our data show that the TT-specific cellular response (TT-specific swelling) is more sensitive to estrogen-mediated suppression than TT-specific IgG1 production.

### ERA-63 inhibits the tetanus toxoid (TT)-specific DTH response in WT and ERβ-/- mice but not in ERα-/- mice

**Figure 2:**
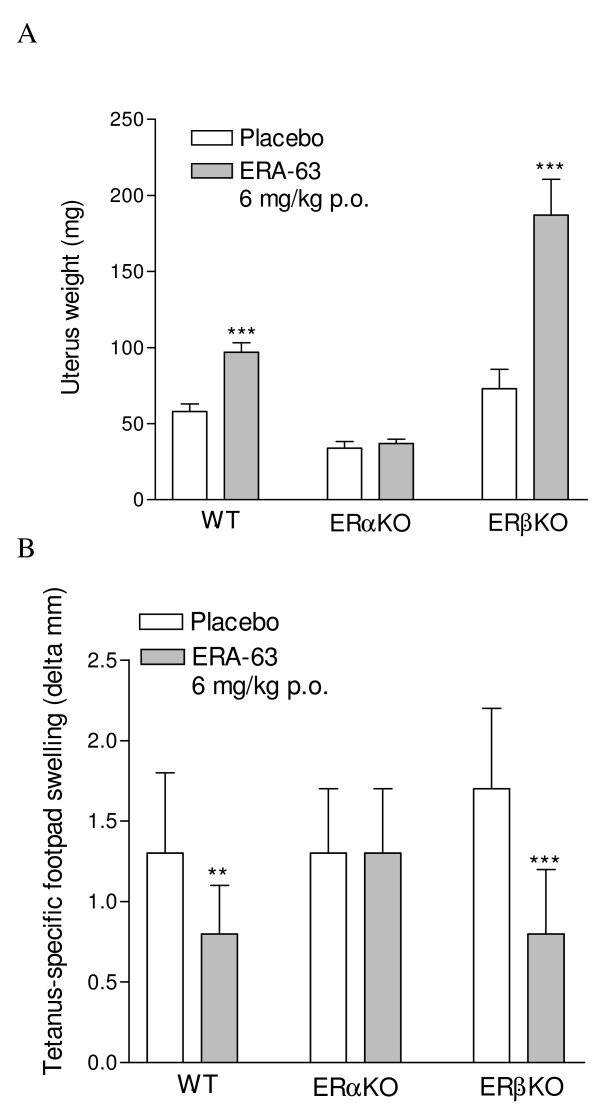
**ERA-63 inhibits the tetanus-specific delayed type hypersensitivity response in WT and ERβKO but not in the ERαKO mice**. Mice were ovariectomized at day -14. Mice (n = 8 per group) were orally treated daily with ERA-63 at 6 mg/kg or vehicle gelatin-mannitol from day -1 to day 9. At day 0, animals were immunized with tetanus toxoid (TT) in dimethyl dioctadecyl ammonium bromide. At day 7, animals were challenged with TT in Al(OH)_3_. The left control footpad received vehicle only. **(A) **At autopsy (forty eight hours later) the uterus was removed and weighed. **(B) **Twenty-four hours later the left and right hind footpad thickness was measured and the delta mm of antigen-specific footpad swelling was calculated according to the following formula: [(swelling left (mm) minus swelling right (mm)]. Data are representative for three independent experiments. Statistical analysis was performed with the analysis of variance test. ** *P *≤ 0.01, *** *P *≤ 0.001. ER, estrogen receptor; WT, wild type.

To further substantiate our findings on ERα-mediated immune suppression, we evaluated ERA-63 on suppression of the TT-specific DTH in wild type, ERα-/- and ERβ-/- mice. Again, we used the uterus weights as a PD marker for the classic estrogenic effect. ERA-63 when tested at one, relatively high, daily, dose of 6 mg/kg, increased the uterus weights in wild type C57bl/6 mice and in ERβ-/- but not in ERα-/- mice, thereby providing further evidence that the increase in uterus weight is indeed mediated via ERα (Figure 2a). Interestingly, after treatment with ERA-63, a more profound increase in uterus weight was observed in ERβ-/- mice when compared with wild type mice. This may be explained by either an increase in ERα receptor expression in ERβ-/- mice [35] or a lack of ERβ -mediated inhibition of ERα signaling [36].

In wild type, ERα-/- and ERβ-/- mice, a similar level of TT-specific swelling was observed in vehicle-treated mice, which allowed for further compound profiling in this model (Figure 2b). Importantly, treatment with the ERα-agonist ERA-63 decreased the DTH response in both wild type and ERβ-/- but not in ERα-/- mice (Figure 2b). The results confirm that suppression of inflammation *in vivo *is mediated via ERα but not ERβ.

### The estrogen-mediated suppression of inflammation and joint destruction in murine CIA is dependent on ERα

**Figure 3:**
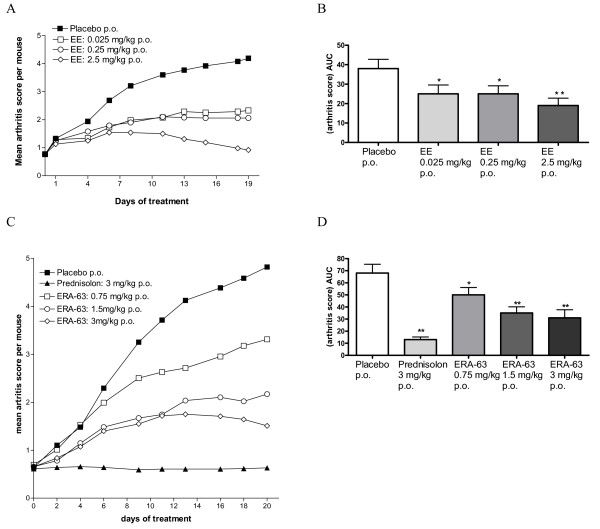
**Suppression of arthritis in collagen-induced arthritis is ERα mediated**. DBA/1J mice were immunized at day 0 and boosted at day 21. Arthritic animals (n = 12 per group) were daily, orally (p.o.) treated with vehicle (gelatin-mannitol), prednisolone (1.5 mg/kg or 3 mg/kg), **(a) **ethinyl estradiol (EE) at (0.025, 0.25 and 2.5 mg/kg) or the **(c) **estrogen receptor (ER)α agonist ERA-63 (0.75, 1.5 and 3 mg/kg). **(a and c) **The severity of arthritis was assessed by visual examination of a total of four paws/mouse (maximum is eight per mouse). The area under the curve (AUC) of the overall arthritis score is computed as a measure for the arthritis severity per animal during the 19 to 21 days of drug treatment for **(b) **EE and **(d) **ERA-63, respectively.

To further confirm that the anti-arthritic properties of estrogens are mediated via ERα, arthritic male DBA/1 mice were orally treated with the ER non-selective estrogen EE, when administered therapeutically in similar doses as used in the TT-DTH. A dose-dependent reduction of disease severity was observed (Figure 3a). When examined by AUC analysis covering the entire treatment period, a significant reduction of the AUC arthritis score was seen (Figure 3b). To determine whether the estrogen-induced immune modulation in this model was indeed mediated through ERα, we used the ERα-agonist ERA-63. Therapeutic administration of the selective agonist ERA-63 decreased the clinical signs of arthritis dose-dependently (Figure 3c). In addition, the AUC analysis over the 20-day treatment period revealed a significant dose-dependent reduction in the ERA-63-treated mice when compared with vehicle control (Figure 3d).

**Figure 4:**
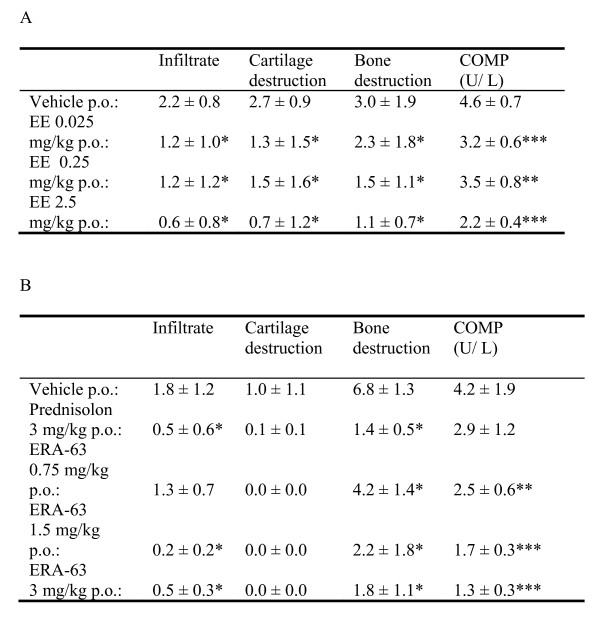
**ERα-mediated suppression of joint destruction**. Arthritic DBA/1J mice (n = 12 per group) were orally (p.o.) treated daily with vehicle (gelatin-mannitol), prednisolone (1.5 or 3 mg/kg), **(a) **ethinyl estradiol (EE) at (0.025, 0.25 and 2.5 mg/kg) or the **(b) **estrogen receptor (ER)α agonist ERA-63 (0.75, 1.5 and 3 mg/kg). At autopsy, knees were evaluated using histopathology (infiltration and cartilage destruction), hind paws were evaluated using X-ray analysis (bone destruction) and serum was used for COMP analysis (cartilage destruction) **(a**, EE) and **(b**, ERA-63). Statistical analyses for COMP and for the area under the curve arthritis score, one-way analysis of variance was used with n = 12 per experimental group (* *P *≤ 0.05).

Histopathological and x-ray analysis of the arthritic joints indicated severe cartilage and bone destruction in the vehicle-treated animals (Figures 4a and 4b). In contrast, EE treatment reduced the amount of inflammatory cells (infiltrate) and measures of cartilage- and bone-destruction significantly. In CIA, serum COMP levels are increased due to enhanced cartilage destruction. Therapeutic treatment with EE decreased the serum COMP (biomarker of cartilage destruction) levels in CIA, which is in line with the protective effect of EE on cartilage destruction as measured with histopathology (Figure 4a). As expected, the ERA-63 suppressed inflammation (AUC) scores in CIA were accompanied by reduced inflammatory infiltrates and cartilage destruction scores at the level of the joint (Figure 4b). The reduced cartilage destruction was associated with a dose-dependent decrease of serum COMP levels. Also, x-ray analysis revealed reduced bone destruction (Figure 4b).

### ERα-mediated immune-suppression is associated with locally decreased IL-1β, IL-6, IL-12p40, KC and RANTES protein levels

It has been reported that CIA is IL-1β and TNFα dependent. Both cytokines have a prominent role in mechanisms underlying joint destruction [32]. Joint synovial cytokines and chemokines were thus measured to evaluate whether the mechanism of ERα-mediated immune-suppression is associated with changed levels of cytokines such as IL-1β. Of 18 different cytokines and chemokines evaluated, EE significantly decreased the amount of IL-1β, IL-6, IL-12p40, KC and RANTES (Table 3). IL-1α, IL-2, IL-3, IL-4, IL-5, IL-10, IL-12p70, IL-17, G-CSF, GM-CSF, IFNγ, MIP-1α and TNFα were not detected in the synovium at this stage of the disease process. Importantly, treatment with the ERα-agonist ERA-63 at higher dosages similarly decreased the level of IL-1β, IL-6, IL-12p40, KC and RANTES, which is in full agreement with the effect of EE treatment (Table 3).

**Table 3:** ERα-mediated suppression of arthritis is associated with decreased cytokine and chemokine levels

	IL-1β (pg/mg protein)	IL-6 (pg/mg protein)	IL-12p40 (pg/mg protein)	KC (pg/mg protein)	RANTES (pg/mg protein)
Vehicle:	91 ± 22	6 ± 3	5 ± 2	17 ± 5	3.2 ± 1.2
EE 0.025 mg/kg po:	13 ± 38*	12 ± 11	1.9 ± 0.5*	5 ± 2	0.9 ± 0.2*
EE 0.25 mg/kg po:	23 ± 18*	1.0 ± 0.4*	1.5 ± 0.1*	5 ± 4	0.8 ± 0.4*
EE 2.5 mg/kg po	4 ± 2*	0.3 ± 0.3*	0.1 ± 0.1*	2 ± 0.3	0.2 ± 0.1*
Vehicle:	110.4	10.7	3.7	19.1	3.3
Prednisolone 3 mg/kg po	0.6	1.3	0.1	0.6	0
ERA-63 0.75 mg/kg po	71.4	21.3	2.2	20.5	1.4
ERA-63 1.5 mg/kg po	5.9	0.7	0.8	1.3	0.2
ERA-63 3 mg/kg po	2.9	2.0	0.6	2.3	0.2

Biopsies of four mice (n = 4 per cage) were pooled and processed for cytokine and chemokine detection, thereby yielding three samples per treatment group for Luminex measurement. Results are presented as mean picograms per mg total protein obtained from three measurements per treatment group in the first experiment comparing vehicle with ethinyl estradiol (EE; 0.025, 0.25 and 2.5 mg/kg). The standard error of the mean are given. Statistical analysis was performed with the two-tailed Mann-Whitney test. Statistically significant differences (*P *≤ 0.05) when compared with the vehicle treatment group is indicated with an asterisk (*). Next, results are based on one measurement on pooled synovial lysates obtained from 12 animals in total (n = 12 mice; 1 biopsy per mouse) in the second experiment comparing vehicle with ERA-63 (0.75, 1.5 and 3 mg/kg). Levels are expressed as picograms per mg total protein. ER, estrogen receptor; po, orally. Chemokine (C-C motif) ligand 5 also known as CCL5 or RANTES and the murine IL-8 homoloque KC.

In summary, our results present a strong case for ERα-mediated suppression of the inflammatory response in rat AA and in established mouse CIA where it is associated with reduced inflammatory cytokine production in the synovium. It remains to be established whether the data in the preclinical models can be translated to the clinical setting.

## Discussion

Our main finding is that estrogen-mediated suppression of inflammation as seen in the TT-DTH response and in experimental arthritis is mediated via ERα but not ERβ.

The mechanisms underlying estrogen modulation of inflammation are not well understood. Both pro-inflammatory and anti-inflammatory effects have been reported (recently reviewed by Straub [20]. The dualistic action of estrogens is, among others, explained by the concentration of circulating estrogen, the differential expression of ERα or ERβ in different cell types and the microenvironment involved impacting the class of the immune response. Here, we focused on defining the contribution of ERα or ERβ to modulation of the inflammatory response in experimental rat and mouse models.

Previously, ERβ-mediated suppression of inflammation in Lewis rat AA was reported [25]. This prompted us to study the effects of EE and our selective ERB-79 in rat AA. Interestingly, our data showed significant suppression of signs and symptoms in rat AA with EE but not with ERB-79, suggesting that in this model the suppression of arthritis is ERα-mediated. Thus, our findings do not confirm the reported effects of an other ERβ agonistic compound (ERB-041) in rat AA [25]. This discrepancy may be explained by a higher potency and selectivity of ERB-79 (484-fold over ERα). The combined data on ERB-79 selectivity, PharmacoKinetics (PK) and the results of the ERα titration study *in vivo *provides evidence that ERB-79 at 3 mg/kg subcutaneous does not demonstrate significant ERα-mediated activity *in vivo*, but is very likely to engage ERβ.

Next, we chose to further study the role of the different ERs in mice *in vivo *using well-described ERα and ERβ knockout mice (compared with wild type) and two highly selective compounds from our compound libraries, which agonistically engage ERα (ERA-63) or ERβ (ERB-79) for cross comparison. This approach was inspired by the notions that: female mice, as seen in the human situation, show pregnancy-associated protection of joint disease with post-partum flares of arthritis [4,5]; and effects of estrogens are best studied *in vivo *representing a system with near physiological levels of ERα and ERβ allowing for ER cross regulation and signaling in context.

Suppressive estrogen effects on DTH responses have been observed previously [37,38]. Also, blocking of ERs by the antagonist ICI 182,780 significantly increased the DTH response [38]. In addition, Islander and colleagues [37] showed that E2 decreased the DTH response in wild type mice whereas this was not seen in ERαβ double knockout mice. These data substantiate the role of estrogens in suppression of the DTH response but do not elucidate the relative roles of ERα or ERβ in this process. Our study is the first to show that treatment with a selective ERα agonist (ERA-63), but not with an ERβ agonist (ERB-79), significantly reduces antigen-specific swelling in the TT-DTH model. This was further confirmed by the use of ERα- and Erβ knockout mice where the ERα agonist ERA-63 decreased the DTH response in both wild type and ERβ^-/- ^but not in ERα^-/- ^mice.

Previous studies have demonstrated effective treatment of inflammation in models of autoimmune disease using estrogens [8-10,25]. Also, estrogens were effective in suppression of joint inflammation and clinical signs of arthritis in mouse and rat CIA [23,24,39,40]. Moreover, ER-receptor blockade using the ER antagonist ICI 182,780 triggered an earlier onset and increased severity of CIA [10]. A number of studies using different selective ER modulators in experimental models of autoimmunity suggest that suppression of inflammation is ERα-mediated rather than ERβ-mediated [41]. Recently, the study by Yh and colleagues showed that estrogen-mediated modulation of inflammatory symptoms in mouse antigen-induced arthritis was ERα-mediated. An ERβ selective compound (8beta-VE2) had no effect in this model [42]. In addition, it has been suggested that ERα, in contrast to ERβ, has a major role in bone homeostasis and therefore may protect against inflammation-induced bone loss [43].

To confirm that estrogen-mediated suppression of inflammation is ERα-mediated in ongoing arthritis, mice with CIA, having scores ranging between 0.25 and 1.25, were treated with EE and ERA-63. ERA-63 strongly suppressed the ongoing arthritic process as evidenced by both a significant reduction of the AUC and a reduction in joint histopathology scores. Moreover, we observed significantly decreased serum COMP levels in the ERA-63 and EE-treated mice. The reductions in COMP levels were associated with prevention of cartilage destruction as evidenced by histopathological examination.

Experimental and clinical studies have established prominent roles for TNFα, IL-6 and IL-1 inflammatory pathways in arthritis. In CIA, an increase in the arthritis score of the knee joints was associated with an increase in IL-1 mRNA levels [33]. In addition, suppression of CIA was observed using antibodies against TNFα and IL-1 [32]. We showed that reduction in symptoms and associated joint pathology by ERA-63 was associated with significantly reduced IL-1β, IL-6, IL-12p40, KC and RANTES protein levels in the synovium. This is in line with previous studies showing estrogen-mediated suppression of nuclear factor (NF) κB activation. It is tempting to speculate that ER cross talk with NFκB may be ligand dependent. Selective ER modulators or ERα-selective ligands may thus have differential effects in different cells. Indeed, E2 was found to suppress NFκB activation whereas the selective ER modulators raloxifene or tamoxifene were inactive in this model system [41].

The role of estrogens in inflammation was recently reviewed [20]. It was proposed, substantiated by numerous studies, that the humoral immune response is stimulated at a broad range of physiologic estrogen concentrations (post-menopausal through to late pregnancy levels) whereas both the innate and the cellular response are suppressed at high physiologic estrogen concentrations (pregnancy levels). This hypothesis would, to a certain extent, explain the higher frequencies of certain autoimmune diseases with a strong B cell component (for example, SLE) in women in the reproductive years. Moreover, it would explain the increase in development of autoimmunity (for example, RA) in menopause when estrogen levels are relatively low.

Our studies unequivocally show that in DTH and in two experimental arthritis models, ERα agonism is needed to suppress the inflammatory response. There is still some controversy around the topic of additional ERs such as GPR30 [44]. Our current study and the study by Engdahl and colleagues confirm the important role of ERα in arthritis suppression and imply that a role for GPR30 in inflammation is not likely [45]. Further studies will be needed to elucidate the relative roles of ERα and ERβ in human autoimmune diseases.

Differential effects of ERα and ERβ ligands in EAE have been described [46,47]. Moreover, clinical trials with oestrogens in multiple sclerosis have been described showing immune modulatory effects [48,49]. Clinical trials involving estrogen suppletion in RA have led to conflicting reports. Early studies, without placebo-controlled treatment groups, demonstrated efficacy of estrogen treatment in RA [50,51]. In placebo-controlled trials, however, different outcomes were documented. Studies with clinical efficacy [52,53] but also studies with marginal [54] or no improvement have been reported [55]. The reasons for the contradictory results on clinical signs in these studies were attributed to selection of the patients, design of the study and the readouts, the power to detect a clinical effect and the use of a combination of estrogens and progestagens, which may obscure effects of estrogen alone [5,56]. Importantly, in several trials, changes in bone formation (osteocalcin) and bone resorption (CTXII) markers were in agreement with favorable effects of estrogens on bone mineral density [54]. Recently, the data from a first proof of concept trial in postmenopausal female RA patients (on concomitant treatment with methotrexate or sulfasalazine) failed to demonstrate efficacy of ERA-63 in spite of good pharmacodynamics [57]. It is feasible that the length of the treatment period (10 weeks) is too short to modulate clinical disease expression under the cover of concurrent treatment. Alternatively, ERα agonism is not beneficial in this group of RA patients. Clearly, further studies are needed to elucidate the relative roles of ERα and ERβ in human autoimmune disease in order to effectively translate this knowledge to novel targeted therapies.

## Conclusions

ERα, but not ERβ, is key in ER-mediated suppression of experimental arthritis. It remains to be investigated how these findings translate to human autoimmune disease.

## Abbreviations

AA: adjuvant arthritis; ANOVA: analysis of variance; AUC: area under the curve; CIA: collagen-induced arthritis; DTH: delayed type hypersensitivity; EAE: experimental autoimmune encephalomyelitis; EE: ethinyl-estradiol; ELISA: enzyme-linked immunosorbent assay; ER: estrogen receptor; IFN: interferon; IL: interleukin; NF: nuclear factor; RA: rheumatoid arthritis; SLE: systemic lupus erythromatosus; TNF: tumor necrosis factor; TT: tetanus toxoid.

## Competing interests

The authors, who are all employees of Schering-Plough, declare that they have no competing interests.

## Authors' contributions

JD designed and supervised the experiments, analyzed data and prepared the manuscript. CvD, DVG and JdG performed the experiments. CLH and FAD helped design the experiments and reviewed the manuscript. PV performed the statistical analysis. AMHB reviewed experimental design, data and prepared the manuscript. All authors have read and approved the final manuscript.
